# Mycorrhiza helper bacteria as stage-specific modulators of fungal development

**DOI:** 10.3389/fpls.2026.1913714

**Published:** 2026-08-25

**Authors:** Alessandro Pennesi, Antonietta Mello, Matilde Acquaviva, Bianca Ranocchi, Antonella Amicucci

**Affiliations:** 1 Department of Biomolecular Science (DISB), University of Urbino, Urbino, Italy; 2 Institute for Sustainable Plant Protection (IPSP), National Research Council (CNR), Turin, Italy

**Keywords:** mycorrhiza helper bacteria, mycorrhizal fungi, rhizosphere, bacteria-fungus interaction, fungal developmental regulation, transcriptional reprogramming

## Abstract

The rhizosphere is a narrow and dynamic soil zone where bacteria and fungi establish symbiotic and synergistic relationships with each other and with plants, collectively influencing nutrient cycling, soil health, and overall ecosystem functioning. Mycorrhiza Helper Bacteria (MHB) are a diverse group of bacterial taxa that promote mycorrhiza establishment, together with those that positively influence already established symbiotic associations. MHB have been largely described from the perspectives of interaction mechanisms, ecological functions, or agricultural applications, treating them as generalized fungal growth-promoters or biofertilizers. However, how their effects change throughout fungal development has received little attention. This review addresses this knowledge gap by reframing MHB not as generic growth promoters, but as stage-specific regulators of the fungal development program. We integrate current evidence on transcriptional responses, metabolite-mediated signaling, and emerging small RNA regulatory networks across five sequential phases of the fungal life cycle: spore germination, presymbiotic hyphal growth, mycelial expansion, root colonization, and nutrient uptake. Elucidating these stage-specific regulatory mechanisms may provide the basis for the rational, stage-targeted design of MHB-based innovative strategies for sustainable agriculture.

## 1 Introduction

The concept was first introduced by Garbaye (1994) under the term “Mycorrhization Helper Bacteria”, describing microorganisms capable of promoting the establishment of symbiotic relationships between plants and fungi (Garbaye, 1994). In subsequent literature, this concept has most commonly been referred to as “Mycorrhiza Helper Bacteria” (Frey-Klett and Garbaye, 2005). Now, the term has acquired a broader meaning, effectively describing any bacterial species yielding a positive influence on fungal growth, stress response, or other metabolic processes (Nasslahsen et al., 2022). This broadening, however, conflates MHB with Plant Growth Promoting Bacteria (PGPB) (Gupta and Chakraborty, 2020): while PGPB act primarily on the plant host, for example, through phytohormone production, nutrient solubilization, or biocontrol activity, MHB specifically target the fungal symbiont, facilitating spore germination, hyphal growth or root colonization by the mycorrhizal fungus. The two groups overlap considerably, since several bacterial taxa display both activities and plant, fungus, and bacterium ultimately form a three-way symbiosis (Garbaye, 1994).

The MHB group comprises a variety of species, including both Gram-positive and Gram-negative bacteria, that appear to be acquired by the fungus directly from the soil during its brief free-living stage before plant-root colonization (Garbaye, 1994). The specificity employed by the fungus in choosing MHB partners is still under investigation; however, some studies propose a metabolic basis for this. Deveau et al. (2010) showed that trehalose, a disaccharide that accumulates inside fungal cells but is not actively released by the fungus, attracts the helper bacterium *Pseudomonas fluorescens* BBc6R8 and supports its growth, forming a trophic mutualism between the *Laccaria bicolor* and the bacterium. Since the fungus does not normally export trehalose, the authors proposed that it becomes available to the bacterium through the natural turnover of hyphal tissue, rather than an active signaling mechanism. Additionally, both symbiotic partners seem to employ a rich repertoire of chemical compounds, excretory systems and secondary metabolites (Hazarika et al., 2023) as a way of communication, possibly affecting the specificity of association (Garbaye, 1994). In this regard, the bacterial quorum sensing (QS) systems, based on diffusible signaling molecules, can create and maintain symbiotic interactions with fungi and represent a common mode of regulatory communication. In *Rhizobium* sp., QS has a fundamental role in nodulation, nitrogen fixation, and symbiosome development, regulating bacterial gene expression collectively (Bonfante and Anca, 2009). Kai et al. (2012) reported a QS system between the zygomycete fungus *Mortierella alpina* and its endobacteria, where the production of bacterial N-acylhomoserine lactones (AHLs) affects the growth and physiology of the fungal mycelium. However, evidence for a functional role of QS in mycorrhizal system remains largely unexplored.

Among chemical signals, communication within mycorrhizal systems can also occur through volatile organic compounds (VOCs), which are released by organisms across all kingdoms and mediate intra- and interspecific interactions both above and below ground (Effmert et al., 2012; Vlot and Rosenkranz, 2022). Within mycorrhizal symbioses, VOCs represent a long-distance signaling mechanism that can modulate different fungal life-cycle stages, including spore germination, mycelial growth and host recognition. Bacterial VOCs have been shown to affect soil fungi, including mycorrhizal species (Mackie and Wheatley, 1999). Garbaye and Duponnois (1993) found that volatiles produced by MHB may either promote or inhibit mycelial growth, depending on the fungal species. In the same way, Aspray et al. (2006) showed that volatiles led to accelerated mycorrhiza development when *Paenibacillus* sp. EJP73, *Lactarius rufus* and *Pinus sylvestris* were in direct contact, but prevented *L. rufus* from forming mycorrhizae with pine seedlings when volatiles were produced by *Paenibacillus* sp. grown away from roots. These contradictory results reflect that VOC-mediated effects are not uniformly beneficial but depend on the spatial and physiological context of the interaction, underscoring that MHB cannot be treated as unconditionally growth-promoting. Bacterial VOCs are also implicated indirectly in the life cycle of truffles: truffle-associated bacteria contribute to the complex aroma of truffles by synthesizing sulphur-containing volatiles such as thiophene derivatives (Splivallo et al., 2015).

MHB exhibit cases of high selectivity, which provides an evolutionary advantage in terms of fitness to associated fungi and plants as well as contributes to the ecological succession of mycorrhizae in aging forests (Duponnois and Garbaye, 1990; Garbaye, 1994). These soilborne symbiotic relationships can be divided into endohyphal, exohyphal or extrahyphal (contactless) main groups, based on the localization of the bacterial partner (Hazarika et al., 2023). With respect to endohyphal associations, however, the molecular and biochemical machinery allowing such communication is deeply dependent on the ability of the bacterium to damage and penetrate the fungal cell wall and is thereby mostly represented by compounds of antibiotic and antimycotic nature, capable of impeding fungal growth and regeneration. Commonly employed enzymes to breach fungal defenses are chitinases and proteases, to threaten fungal structural integrity; lipases and phospholipases, to destabilize cell membranes; and massive production of ROS (reactive oxygen species) causes oxidative damage to the hyphae and facilitates bacterial permeation (Hazarika et al., 2023). Such interactions alter gene expression and, consequently, physiological behaviors, including pathogenicity, sporulation, biofilm production, hyphal growth, and structural modifications (Hazarika et al., 2023). Much of this evidence, however, derives from non-mycorrhizal or facultatively pathogenic fungal systems, and the extent to which endohyphal invasion mechanisms extend to ectomycorrhizal basidiomycetes, which differ in cell wall composition and in the frequency of endobacterial associations, remains to be established.

Thus, MHB impact the fungal life cycle through stage-specific transcriptional reprogramming, modulation of signaling pathways and activation of metabolic processes, although the degree of stage-specificity has rarely been assessed systematically within a single study.

Overall, MHB can modulate fungal growth, morphology, and stress responses through finely tuned regulatory mechanisms, whose intensity and molecular targets appear to shift according to the developmental stage of the fungal partner. In this review, we argue that this stage-dependence, rather than a generalized growth-promoting activity, constitutes the most distinctive and informative feature of MHB-fungus interactions. Accordingly, we integrate molecular, transcriptional and signaling pathways data to describe how MHB influence fungal life-cycle across distinct stages of the fungal life cycle: spore germination, presymbiotic hyphal growth, mycelial expansion, root colonization, and nutrient acquisition. We consider evidence from both arbuscular and ectomycorrhizal systems, and, where relevant, briefly address ericoid mycorrhizal associations. Throughout, we distinguish demonstrated mechanisms from indirect evidence and speculative hypotheses, an aspect we consider particularly important given how often conclusions in this field have relied on extrapolation from non-mycorrhizal model systems. We begin by outlining the evidence for MHB activity across mycorrhizal systems, before turning to the stage-specific mechanisms underlying fungal development regulation, from presymbiotic signaling to root colonization and nutrient acquisition, and finally considering their ecological and applied implications, including for sustainable agriculture. By focusing on stage-dependence and mechanistic insights, we aim to provide a more precise conceptual framework for future research on fungal-bacterial symbiosis.

## 2 Evidence of mycorrhiza helper bacteria

Ever since Mosse (1962) isolated the first recorded MHB strain from arbuscular mycorrhizal fungi (AMF) belonging to the genus *Glomus*, numerous bacterial species and genera have been identified as putative mycorrhizal partners (Frey‐Klett et al., 2007). Given that most terrestrial plants depend on mycorrhizal fungi to survive and thrive, the ecological significance of this specific bacterial group is paramount. Beneficial effects of MHB have been observed both in AMF and ectomycorrhizal fungi (EcMF) (Frey‐Klett et al., 2007). The former includes species belonging to the phylum *Glomeromycota* (in recent years, this has been reclassified as the subdivision *Glomeromycotina* within the phylum *Mucoromycota*) and represents the most widespread plant-fungal association. The latter is a less widespread form of symbiosis, associated with fungi belonging to the taxa *Basidiomycetes* and *Ascomycetes* (Gupta and Chakraborty, 2020). From the ecological point of view, AMF are primarily involved in the transport of phosphorus to the host plants but, lacking key enzymes for degrading complex organic matter, they are unable to directly utilize organic nutrients from the soil. This feature pushes these fungi to form close interactions with MHB, which can solubilize phosphorus and other elements (Duan et al., 2024). In contrast to AMF, ECM have some saprotrophic activities, being able to degrade organic matter through their set of extracellular enzymes (Shah et al., 2016). Despite this capability, they still need the help of MHB to mobilize nutrients such as nitrogen, which is often a primary limiting factor in boreal and temperate forests, and phosphorus under stress or nutrient-poor conditions (Guan et al., 2026). A wide taxonomic variety of MHB has been successfully isolated from arbuscular mycorrhizae: the most abundant genera being *Achromobacter*, *Bacillus*, *Pseudomonas*, *Sphingomonas*, *Paenibacillus*, *Arthrobacter*, *Rhizobium*, *Streptomyces*, *Variovorax*, *Lysobacter*, *Burkholderia*, *Chryseobacterium*, *Leifsonia*, *Massilia*, and *Microbacterium*, as well as species from *Burkholderiaceae* (*Proteobacteria*) and *Mycoplasmataceae* (*Tenericutes*) families (Basiru et al., 2023). Functionally, these bacterial taxa are primarily implicated in nutrient uptake, hormone production and promotion of fungal infection, thus actively aiding the establishment of the plant-fungus symbiosis and sustaining fungal growth (Basiru et al., 2023). Regarding ECM, early literature suggested that only *Basidiomycetes* could recruit MHB as symbionts to promote mycorrhizal activity (Frey‐Klett et al., 2007). Recent studies show that several *Ascomycete* species also benefit from the interaction with soil bacteria (Reis et al., 2021). The prevalent bacterial genera involved are *Arthrobacter*, *Bacillus*, *Bradyrhizobium*, *Burkholderia*, *Pseudomonas*, *Rhizobium*, and *Streptomyces*, mainly acting as symbiotic enhancers or nutrient providers to ectomycorrhizae (Reis et al., 2021).

## 3 Influence of MHB at different stages of the fungal life cycle

**Figure 1:**
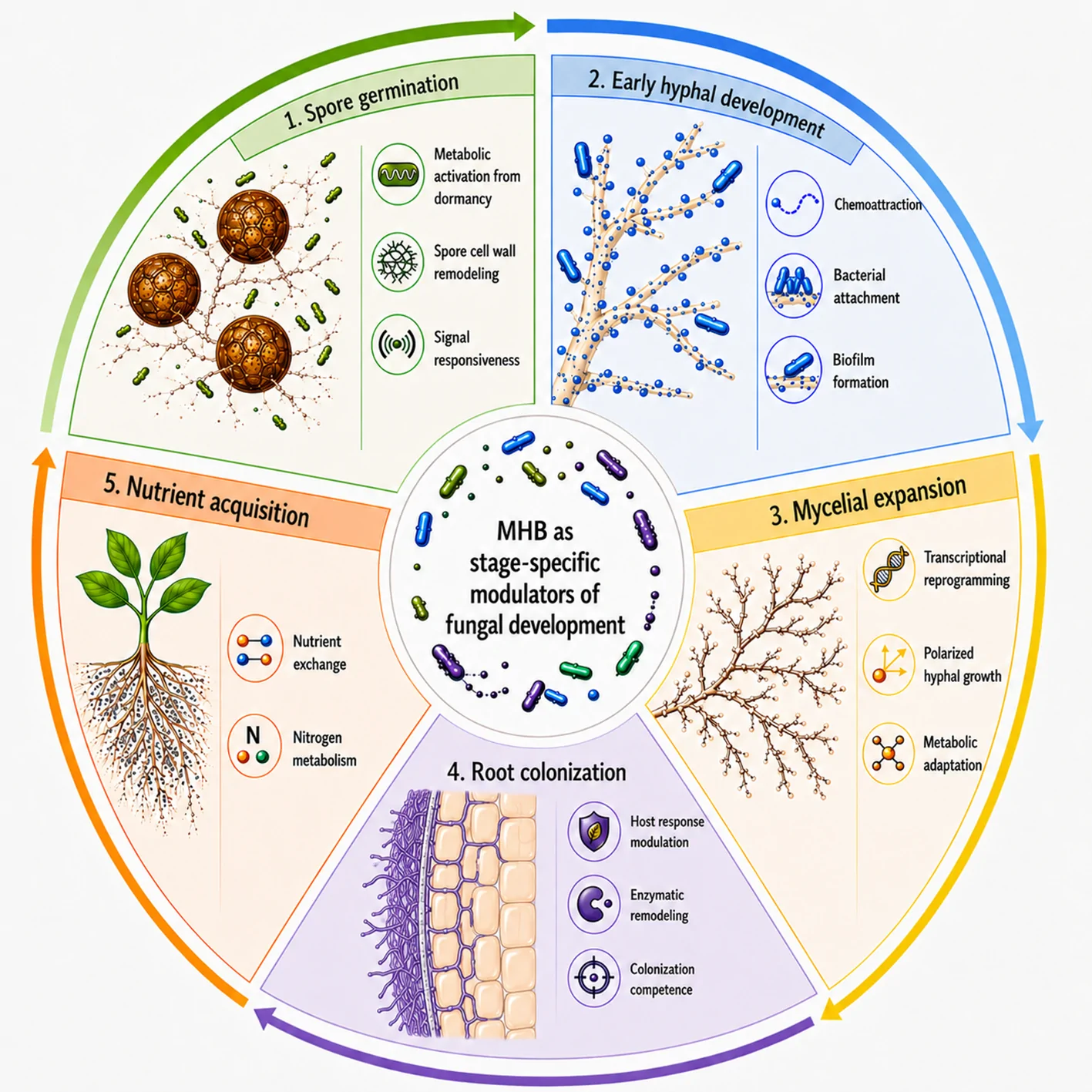
Stage-specific modulation of fungal development mediated by Mycorrhiza Helper Bacteria (MHB). Schematic representation of fungal developmental stages influenced by MHB, including spore germination, early hyphal development, mycelial expansion, root colonization and nutrient acquisition. MHB coordinate distinct biological processes at each developmental stage. Rather than acting as generic fungal growth promoters, MHB exert a stage-dependent impact that collectively supports fungal development. Overall, MHB contribute to improving fungi and plant productivity, increasing stress tolerance and supporting sustainable agricultural practices. The detailed mechanisms promoted by MHB at each fungal phase are reported in the corresponding section of the text.

Since the term MHB is currently used to describe bacterial taxa that promote both the establishment of mycorrhizal symbiosis and the functioning of already established associations, these two functional categories may be represented by distinct or partially overlapping microbial groups (Nasslahsen et al., 2022). Rather than acting through a single conserved mechanism, the impact of MHB on fungal development is dynamic and occurs during different stages of its life cycle. Their effects depend on the fungal host, the bacterial partner, and the surrounding environmental conditions. Accordingly, MHB modulate fungal physiology through complementary mechanisms, including transcriptional reprogramming, diffusible metabolites and VOCs, enzymatic activities, and emerging small RNA regulatory networks. The following sections summarize how MHB, through these mechanisms, contribute to successive fungal life stages, from spore germination and presymbiotic growth to mycelial expansion, root colonization, and nutrient acquisition (**Figure 1**). Addressing the knowledge gaps regarding the MHB influence on fungal development throughout the life cycle stages is essential for clarifying their ecological role and supporting the rational development of MHB-based strategies for sustainable agriculture and forestry.

### 3.1 The role of MHB in spore germination

Spore germination represents the initial and most critical phase of fungal growth, representing the transition from dormancy to active growth. Although this step is not directly regulated by host plant signals, it is strongly influenced by physical, chemical and microbiological conditions of the surrounding soil (Giovannetti, 2000). Confocal and electron microscopy analyses have shown that fungal spores can act as niches for microbial communities (Desirò et al., 2014), reinforcing the concept that bacteria are structurally integrated within the fungal propagule rather than passive colonizers. Within this early developmental context, MHB modulate both the initiation of germination and the metabolic activation required for subsequent hyphal development (**Table 1**). The following sections summarize the current evidence indicating that bacterial effects on spore germination rely on complementary mechanisms, including chemical signaling, enzymatic remodeling of the spore cell wall and transcriptional reprogramming of fungal metabolism.

**Table 1:** Impact of MHB on spore germination.

Mycorrhizal fungi	Mycorrhizal type	Bacteria involved	Modalities of interaction	Reported effects	References
*Glomus mosseae*	AMF	Rhizosphere bacteria (unidentified)	Culture filtrates	Stimulation of chlamydospores	(Azcón, 1987)
*Basidiomycetes*	AMF	*Corynebacterium, Pseudomonas* spp.	Diffusible metabolites	Stimulation of basidiospore germination	(Ali and Jackson, 1989)
*Glomus* spp.	AMF	*Streptomyces* spp.	Diffusible metabolites	Stimulation of spore germination	(Tylka, 1991)
*Tuber* spp.	EcMF	*Pseudomonas* spp.	Enzymatic activity	Induction of spore germination and ascospore release through cellulolytic and chitinolytic activities	(Citterio et al., 2001; Pavić et al., 2013; Monaco et al., 2022)
*Glomus clarum* (*Rhizophagus clarus)*	AMF	*Bacillus pabuli* LA3	Culture filtrates	Stimulation of spore germination and subsequent hyphal growth	(Xavier and Germida, 2003)
*Glomus clarum (Rhizophagus clarus*)	AMF	*Bacillus chitinosporus* LA6a, *Bacillus laterosporus* LA6b	Culture filtrates	Inhibition of spore germination	(Xavier and Germida, 2003)
*Gigaspora margarita*	AMF	*Candidatus Glomeribacter gigasporarum*	Transcriptional and metabolic reprogramming	Enhanced metabolic activation during germination	(Salvioli et al., 2016)
*Funneliformis caledonium, Racocetra alborosea* and *Funneliformis mosseae*	AMF	*Candidatus Glomeribacter gigasporarum*	Enzymatic activity	Remodeling of spore walls by cellulases, chitinases, proteases, and enzymes capable of degrading exopolysaccharides (EPS)	(Selvakumar et al., 2016; Long et al., 2017)
*Rhizophagus intraradices*	AMF	*Bacillus simplex, Bacillus megaterium, and Flavobacterium ginsengisoli*	Co-inoculation (consortium); enzymatic activity	Hormone/cell-wall-softening enzyme secretion (*B. simplex*), siderophore production and organic matter decomposition (*F. ginsengisoli*), phosphate solubilization and IAA synthesis (*B. megaterium*)	(Guan et al., 2026)

Experimental evidence describing the effects of MHB on fungal spore germination. The table summarizes the mycorrhizal fungal species, bacterial partners, interaction modalities, reported biological effects, and the corresponding references.

#### 3.1.1 Chemical signaling and enzymatic activity

In AMF, Azcón (1987) was among the first to report that rhizosphere bacteria and their culture filtrates enhanced the germination of *Glomus mosseae* chlamydospores compared to axenic conditions. However, the active compounds were not chemically identified. Accordingly, subsequent studies demonstrated that diffusible metabolites or signaling molecules produced by *Glomus*-associated bacteria can promote spore germination (Hildebrandt et al., 2002, 2006; Xavier and Germida, 2003). Xavier and Germida (2003), isolated bacteria naturally associated with *Glomus clarum* spores and showed that the stimulation of spore germination and subsequent hyphal growth by *Bacillus pabuli* LA3 required direct contact or diffusible non-volatile metabolites. In contrast, inhibition by *Bacillus chitinosporus* LA6a and *Bacillus laterosporus* LA6b was observed in both direct and volatile assays, suggesting that diffusible and volatile bacterial compounds may contribute to inhibitory effects, although these were not chemically identified (Xavier and Germida, 2003). Similarly, selected *Streptomyces* species were shown to induce the germination of AMF spores belonging to the genus *Glomus* under sterile conditions without requiring direct contact, further supporting the involvement of diffusible bacterial factors (Tylka, 1991). Guan et al. (2026) reported that a consortium of MHB significantly promoted the spore germination of *Rhizophagus intraradices*, highlighting their role in facilitating the transition from fungal dormancy to active growth and the subsequent establishment of mycorrhizal symbiosis. Taken together, these early studies indicate that MHB modulate spore germination through multiple modalities, direct contact-dependent signals, diffusible non-volatile metabolites, and VOCs. Importantly, they also highlight a strict bacterial specificity toward the fungal counterpart, since different isolates associated with the same fungal species can either promote or inhibit germination. However, while phenotypic stimulation or inhibition was consistently reported, the fungal molecular targets remained largely unexplored.

Beyond diffusible and volatile compounds, bacteria belonging to the phyla *Proteobacteria*, *Bacteroidetes*, *Firmicutes* and *Actinobacteria* embedded within the spore walls of *Funneliformis caledonium*, *Racocetra alborosea* and *Funneliformis mosseae*, have been shown to produce cell wall–degrading enzymes including cellulases, chitinases, proteases, and exopolysaccharide (EPS)-degrading enzymes (Selvakumar et al., 2016; Long et al., 2017).

A recent viewpoint article focusing on EcMF proposes that bacterial effects on spore germination depend on the physiological competence of fungal spores, whose developmental state may differ within the same spore population. Temporal synchronization between bacterial activity and fungal spore receptivity creates an interaction window during which bacterial communities can influence the transition from dormancy to germination (Berrios, 2025). Moreover, depending on the host tree species and the environmental context, the role of bacteria in spore germination may range from stimulatory to inhibitory (Berrios, 2025). Ali and Jackson (1989) reported that the spore germination of several *Basidiomycetes* was stimulated by *Corynebacterium* and *Pseudomonas stutzeri*, suggesting the involvement of unidentified diffusible bacterial factors. Notably, in the context of hypogeous ECM, Pseudomonas spp. facilitate *Tuber* spore germination and ascospore release through cellulolytic and chitinolytic activities (Citterio et al., 2001; Pavić et al., 2013; Monaco et al., 2022).

Taken together, these findings indicate that MHB promote fungal spore germination through complementary mechanisms, including chemical signaling and bacterial enzymatic modification of the spore cell wall, thereby facilitating germination in both AMF and ECM fungi. However, while these studies clearly demonstrate that MHB-derived signals and enzymes regulate the initiation of fungal germination, they provide only limited insight into the molecular responses induced within spores. This intracellular transcriptional and metabolic reprogramming is discussed in the following section.

#### 3.1.2 Transcriptional and metabolic activation from dormancy

In the case of AMF, the presence of endobacteria appears to significantly contribute to fungal fitness during the presymbiotic stage (Salvioli et al., 2016). Three years of observations showed that *Gigaspora margarita* harboring *Candidatus Glomeribacter gigasporarum* (*CaGg*) produced over 50% more spores compared to cured lines. To evaluate whether host plant-derived signals induce overlapping transcriptional responses, Salvioli et al. (2016) treated fungal spores with the synthetic strigolactone GR24, a well-known pre-symbiotic branching factor in AMF. The comparable transcriptional responses observed following *CaGg* presence and GR24 treatment suggest a partial overlap in the fungal transcriptional profile activated by endobacteria and plant-derived signals, suggesting a convergence of activity by bacterial and plant signals during this phase. At the molecular level, during the germinating spore stage, *CaGg* affected the transcriptional profile of *G. margarita*, upregulating genes involved in primary metabolism, such as glycolysis, gluconeogenesis, the TCA cycle, galactose metabolism, fatty acid degradation, and purine, pyrimidine and amino acid metabolism. This coordinated activation of primary metabolic pathways indicates that endobacteria promotes rapid metabolic activation from dormancy, equipping the germinating spore with enhanced biosynthetic and energetic capacity. In fact, one of the most remarkable transcriptional changes concerned oxidative phosphorylation. Mitochondrial genes, including cytochrome c oxidase subunit 1 (Complex IV), NADH dehydrogenase subunits (Complex I), and NADH-ubiquinone oxidoreductase subunits, were upregulated, indicating an increased capacity for ATP production. Transmission electron microscopy (TEM) further revealed mitochondrial morphological alterations in the absence of the endobacterium, supporting a functional role in energy homeostasis. The strigolactone treatment induced a similar transcriptional response, reinforcing the idea of shared signalling pathways. The transcriptional profile of ROS-scavenging genes was also significantly affected. Thioredoxin reductase, peroxiredoxins, glutathione peroxidase, and Cu/Zn superoxide dismutase were upregulated in the presence of *CaGg*, while selenocompound metabolism, implicated in antioxidant functions, was upregulated following GR24 treatment (Salvioli et al., 2016). These findings suggest that enhanced oxidative phosphorylation is linked with reinforced antioxidant machinery, preventing oxidative stress during the metabolically demanding germination process.

The endobacteria also affected membrane and cell wall plasticity through the upregulation of chitin synthase transcripts and the downregulation of putative chitin deacetylase transcripts in the fungal host.

Since chitin deacetylase catalyzes the conversion of chitin into chitosan (El Gueddari et al., 2002), its downregulation alters cell wall composition and structural properties, which may increase chitin deposition and modify mechanical properties. This transcriptional regulation potentially influences the germinating spore and may facilitate the transition toward the next steps of development. Furthermore, *CaGg* influenced the expression of genes involved in nutrient transport. A plasma membrane iron permease containing the FTR1 domain was among the most upregulated genes both in *CaGg* containing spores and in GR24 treated spores. A high-affinity phosphate transporter belonging to the major facilitator superfamily was also strongly upregulated, whereas some putative ammonium transporters were downregulated. These results indicate that endobacteria modulate the nutrient acquisition strategy of the germinating spore, potentially enhancing its competitive fitness in the rhizosphere before contact with the host plant.

Salvioli et al. (2016) also examined intracellular calcium homeostasis, showing that basal calcium levels were significantly increased in the absence of the endobacterium. Low basal calcium concentrations are essential for proper signaling responsiveness (Case et al., 2007); consequently, its elevated levels may compromise signal perception. These observations suggest that endobacteria help to preserve intracellular signaling competence, potentially facilitating the accurate perception of environmental and host-derived signals during early developmental stages.

Overall, the available evidence indicates that MHB promote the transition from fungal spore dormancy to germination and subsequent active growth through a transcriptional regulation of fungal genes involved in primary metabolism, oxidative phosphorylation, antioxidant machinery, cell wall plasticity and intracellular signaling pathways.

### 3.2 Influence of MHB on early hyphal development

The induction of spore germination is followed by presymbiotic hyphal development, a phase during which the fungus establishes the first physical and molecular interactions with compatible bacterial partners before the next stages of development and symbiosis. Rather than simply promoting hyphal elongation, MHB establish a structured and dynamic link with fungal partners that integrates physical attachment through biofilm formation, effector secretion, transcriptional regulation, diffusible signaling molecules, and emerging regulatory RNA networks (**Table 2**). While these processes configure the physical and signaling connection during early hyphal development, the following section focuses on how these early interactions are translated into sustained mycelial expansion.

**Table 2:** Impact of MHB on early hyphal development.

Mycorrhizal fungi	Mycorrhizal type	Bacteria involved	Modalities of interaction	Reported effects	References
*Laccaria bicolor*	EcMF	*Pseudomonas fluorescens* BBc6R8	Physical attachment and biofilm formation	Surface colonization of fungal hyphae and establishment of structured bacterial-fungal interfaces	(Sen et al., 1996; Frey-Klett et al., 1999)
*Laccaria bicolor*	EcMF	*Pseudomonas fluorescens* BBc6R8	Chemoattraction	Chemoattraction and bacterial recruitment to the hyphal surface	(Frey‐Klett et al., 2007)
*Amanita muscaria*	EcMF	*Streptomyces* sp. AcH505	Diffusible compounds	Actin cytoskeleton reorganization and modulation of fungal polarity	(Schrey et al., 2007)
*Mucor irregularis* SS7	SAP^*^	*Serratia marcescens* D1	Secretion systems	Endohyphal invasion and local fungal cell wall remodeling through secretion systems type VI (T6SS)	(Hazarika et al., 2020)

*SAP, saprophytic fungi.

Experimental evidence describing the effects of MHB on early hyphal development. The table summarizes the mycorrhizal fungal species, bacterial partners, interaction modalities, reported biological effects, and the corresponding references.

#### 3.2.1 Recognition and recruitment

Following spore germination, the emerging hyphae recruit compatible bacterial partners before stable physical associations can be established. Mycorrhizal fungi release a variety of diffusible compounds, including sugars and organic acids, that establish chemical gradients in the hyphosphere, providing carbon and energy sources for bacterial growth while contributing to the enrichment of MHB populations (Guan et al., 2026). Among the compounds proposed to mediate this process, trehalose has received particular attention. In the ectomycorrhizal fungus *L. bicolor*, trehalose accumulates at high concentrations within fungal cells (Martin et al., 1984) and may be released by dead or damaged hyphae (Deveau et al., 2010). In particular, this disaccharide has been shown to chemoattract the helper bacterium *P. fluorescens* BBc6R8 and may promote bacterial accumulation on the hyphal surface and subsequent biofilm formation (Frey‐Klett et al., 2007). Because trehalose is abundant in several ectomycorrhizal fungi, although direct evidence remains limited, this mechanism may extend beyond the *P. fluorescens* BBc6R8–*L. bicolor* system and represent a more general strategy through which fungi recruit beneficial bacterial partners before physical interaction is established. Collectively, these findings suggest that fungal-derived molecules, including trehalose, contribute to the selective recruitment of compatible bacterial partners, establishing the conditions for subsequent bacterial attachment and biofilm formation along the hyphal surface. This interkingdom chemical dialogue represents the first step toward a more stable and synergistic phase.

#### 3.2.2 Physical interaction

The ability of MHB to form biofilm-like structures is required to establish stable physical attachment on the surface of emerging fungal hyphae. During biofilm formation, bacterial cells are embedded within a self-produced extracellular polymeric matrix that promotes stable colonization of the hyphal surface and facilitates long-term bacteria–fungus interactions (Cancino-Diaz et al., 2023; Frey‐Klett et al., 2007). Besides providing structural stability, biofilms create a protected microenvironment that spatially organizes interkingdom communication and may protect bacteria from environmental fluctuations. Direct evidence for biofilm establishment on mycorrhizal hyphae was provided by Sen et al. (1996), who demonstrated that *P. fluorescens* BBc6R8 attaches to the hyphae of several ectomycorrhizal fungi and subsequently develops biofilm-like structures on *L. bicolor* hyphae *in vitro*. This spatial association is consistent with previous observations indicating that BBc6R8 decreases in bulk soil while concentrating on specific niches such as the fungal cell wall (Frey-Klett et al., 1999).

Regarding physical interactions between the two partners, the precise mechanisms of surface attachment are still not fully elucidated; nevertheless, several regulatory pathways controlling biofilm establishment and bacterial colonization of the fungal hyphae have recently begun to emerge.

#### 3.2.3 Emerging regulatory mechanisms

The establishment and maintenance of stable bacteria–fungus associations rely on coordinated regulatory pathways that control bacterial attachment, biofilm development and communication with the fungal host. Among these, cyclic di-guanosine monophosphate (c-di-GMP) acts as a central bacterial second messenger regulating the transition from motile to sessile lifestyles through the control of exopolysaccharide production, surface adhesion and biofilm maturation (López-Baena et al., 2016; Pérez-Mendoza and Sanjuán, 2019; Ainelo et al., 2017). Protein secretion systems represent another emerging component of bacterial–fungal interactions. The activation of bacterial secretion systems (T1SS-T7SS), coupled with chitinase activity, has been proposed to facilitate bacterial colonization and interaction with the fungi (Napitupulu, 2025). Bacterial genes such as gspD (general secretion pathway protein D), secB (protein export chaperone SecB), and components of the type III secretion system (T3SS) are highly expressed during association with fungal hyphae (Pa et al., 2025). In addition, functional studies in endofungal model systems further showed that the type II secretion system (T2SS) mediates localized fungal cell wall remodeling by releasing chitinases, thereby allowing bacterial entry while preserving hyphal integrity (Moebius et al., 2014). In another study, the significant upregulation of two T6SS-associated genes (*TssJ* and an outer membrane murein lipoprotein gene) during the interaction between the endobacterium *Serratia marcescens* D1 and its fungal host *Mucor irregularis* SS7 was proposed to indicate a possible role for this secretion system in the invasion process, although the authors noted that mutation-based approaches are still needed to confirm this functional involvement (Hazarika et al., 2020). It is important to note that the molecular mechanisms described above have been characterized in non-mycorrhizal and, in some cases, pathogenic or antagonistic, endofungal systems. Differences in fungal cell wall composition, interaction biology, and the frequency of endobacteria caution against direct extrapolation of these mechanisms to MHB systems. Thus, further studies specifically addressing secretion systems in MHB-AMF/EcMF interactions are needed to confirm whether similar mechanisms occur. Collectively, these observations support the hypothesis that bacterial secretion systems contribute to intimate bacterial-fungal contact, although direct evidence of their modulation of specific fungal cellular processes and the identity of their fungal targets remain to be established. In addition to protein-mediated regulatory pathways during the early stages of interactions, microbial small noncoding RNAs (sRNAs) have recently emerged as an additional regulatory layer. Regulatory sRNAs enable rapid reprogramming of gene expression in response to environmental variability and contribute to microbial adaptation within host-associated habitats (Huang et al., 2019). Cross-kingdom transfer of sRNAs has been described in symbiotic and pathogenic systems, where they act as effector molecules modulating host gene expression to facilitate successful invasion (Weiberg et al., 2015; Weiberg and Jin, 2015). Despite that, similar mechanisms have not yet been directly demonstrated in helper bacteria–fungus interactions. In *Pseudomonas* spp., the regulatory sRNAs *RsmY* and *RsmZ*, acting downstream of the GacS/GacA two-component system, regulate biofilm formation, motility and host-associated lifestyles (Wolska et al., 2016; Upadhyay et al., 2017). Although these regulatory pathways are likely to influence bacterial adaptation during fungal colonization, their specific role in MHB-mediated interactions has not yet been experimentally demonstrated. Consequently, given the frequent occurrence of *Pseudomonas* spp. in the mycorrhizal microbiome (Pandit et al., 2022), whether bacterial sRNAs participate in interkingdom communication with mycorrhizal fungi remains an open question deserving future investigation.

### 3.3 MHB sustain mycelial expansion

Following presymbiotic activation, the mycelium, the vegetative body of fungi, expands primarily through polarized hyphal tip growth, a process driven by localized cell wall remodeling, targeted exocytosis, and the coordinated uptake of nutrients (Napitupulu, 2025). Continuous hyphal branching subsequently generates an intricate mycelial network that is essential for fungal fitness and the establishment of a functional symbiosis with the host plant. Most of these interaction patterns have been characterized by using *in vitro* dual culture assays, in which bacteria and fungi are grown together under controlled conditions to evaluate their interactions. These assays have been widely employed to investigate principally the direct effects of MHB on macro-morphological changes in hyphal architecture (Frey‐Klett et al., 2007; Deveau and Labbé, 2016).

The following sections summarize how MHB sustain mycelial expansion through coordinated mechanisms involving diffusible metabolites, volatile organic compounds, and transcriptional reprogramming of fungal developmental profile (**Table 3**). Although MHB are commonly identified by their ability to stimulate fungal growth, this response is not universal. The outcome of the interaction largely depends on the identity of both the fungal and bacterial partners. For instance, Schrey et al. (2005) demonstrated that the same *Streptomyces* strain AcH505 exhibited contrasting responses among ectomycorrhizal fungi, promoting mycelial growth in some species while showing no significant effect on *Paxillus involutus* and inhibiting *Hebeloma cylindrosporum*. Similarly, Deveau et al. (2007) reported that, among several bacterial strains tested with *L. bicolor*, only a subset enhanced mycelial growth, whereas other MHB displayed neutral or inhibitory effects under identical *in vitro* conditions.

**Table 3:** Impact of MHB on mycelial growth.

Mycorrhizal fungi	Mycorrhizal type	Bacteria involved	Modalities of interaction	Reported effects	References
*Amanita muscaria*	EcMF	*Streptomyces* sp. AcH505	Transcriptional reprogramming	Enhanced membrane biogenesis, polarized growth, cytoskeletal remodelling and translational activation associated with hyphal expansion	(Schrey et al., 2005)
*Amanita muscaria*	EcMF	*Streptomyces* sp. AcH505	Diffusible metabolite	Improved mycelial growth and ectomycorrhiza formation mediated by auxofuran	(Riedlinger et al., 2006)
*Laccaria bicolor* S238N	EcMF	*Pseudomonas fluorescens* BBc6R8	Transcriptional reprogramming	Modulation of stress responses, energy metabolism, cytoskeleton organization and carbon utilization during prolonged bacterial interaction	(Deveau et al., 2007)
*Pleurotus* spp*, Lactarius rufus*	SAP, EcMF	*Paenibacillus peoriae M48F*	VOC	Enhanced hyphal extension and branching mediated by 2,5-diisopropylpyrazine	(Aspray et al., 2013; Orban et al., 2023)
*Laccaria bicolor* S238N	EcMF	*Pseudomonas fluorescens* BBc6R8	Diffusible metabolite	Enhanced mycelial growth mediated by thiamine (vitamin B1)	(Deveau et al., 2010)

Experimental evidence describing the effects of MHB on mycelial growth. The table summarizes the mycorrhizal fungal species, bacterial partners, interaction modalities, reported biological effects, and the corresponding references.

#### 3.3.1 Fungal transcriptional reprogramming

Evidence of fungal growth stimulation induced by MHB through coordinated transcriptional reprogramming was provided by two pioneering studies, which employed complementary experimental approaches. Schrey et al. (2005) investigated the transcriptional response of *Amanita muscaria* when exposed to diffusible compounds released by *Streptomyces* sp. AcH505 in a contact-independent dual culture system. Instead, Deveau et al. (2007) monitored the temporal dynamics of gene expression in *L. bicolor* through successive stages of interaction with *P. fluorescens* BBc6R8 in a dual culture assay. Despite these methodological differences, both studies converge in demonstrating that MHB activate common biological processes that collectively sustain fungal growth and mycelial expansion. The earliest transcriptional responses are associated with fungal perception and developmental transition before direct attachment occurs. Deveau et al. (2007) showed that, before physical contact, *L. bicolor* upregulated genes encoding Tectonin II, a putative recognition-related protein and *cipc*, a marker of the presymbiotic state (Morel et al., 2005). Concomitantly, genes associated with chromatin organization and transcriptional regulation were induced, **s**uch as those encoding histone H4, Tra1, a component of the SAGA transcriptional regulatory complex (Allard, 1999; Wu et al., 2004) and the splicing factor 3b, together with members of the major facilitator superfamily (MFS). These findings suggest that, before physical contact, bacterial perception rapidly reorganizes the fungal transcriptional machinery. During this early phase, genes involved in coenzyme A biosynthesis (pantothenate kinase) and β-oxidation (fox2) were transiently repressed, also indicating an early redistribution of lipid metabolism as the fungus prepares for active growth (Requena et al., 1999; Deveau et al., 2007). Following bacterial perception, transcriptional regulation shifts toward the activation of processes supporting hyphal extension and cellular remodeling. In the contact-independent system developed by Schrey et al. (2005), diffusible bacterial metabolites induced expression of *AmCyp40* involved in protein folding and stress response; *AmAacs* encoding acetoacetyl-CoA synthetase, a key enzyme in sterol biosynthesis and ergosterol accumulation; *Tpk3* encoding the catalytic subunit of protein kinase A involved in cAMP-dependent signaling, and a gene encoding a Don3-like p21-activated kinase associated with cytoskeletal reorganization. Induction of sterol biosynthesis-related pathways suggests reinforcement of membrane biogenesis, an essential process supporting sustained hyphal extension. In addition, genes involved in protein synthesis and translational capacity, including ribosomal proteins and N², N²-dimethylguanosine tRNA methyltransferase, were also upregulated, collectively supporting a global activation of fungal growth (Schrey et al., 2005). Comparable functional responses were observed at the time of physical contact in *L. bicolor*, where induction of a heat shock protein and a protein kinase reflected increased protein folding requirements and activation of signaling pathways associated with fungal morphogenesis. Conversely, two glutathione S-transferase genes involved in detoxification were transiently downregulated, suggesting that the establishment of a compatible bacterial–fungal association reduces the requirement for stress-associated defense responses (Deveau et al., 2007).

As the interaction progressed, fungal gene expression was characterized by metabolic adaptation associated with maintenance of the established bacterial association. Deveau et al. (2007) reported coordinated repression of genes involved in protein synthesis and turnover, including several ribosomal proteins, the translation initiation factor SU1, ubiquitin-related proteins and actin-1, indicating attenuation of protein synthesis, cytoskeletal remodeling and proteome turnover once active hyphal development has been established. Likewise, genes associated with mitochondrial energy metabolism, including ATP synthase subunits, ADP/ATP carrier proteins, malic enzyme, phosphoenolpyruvate carboxylase and fumarate reductase, were downregulated, whereas citrate synthase was induced, suggesting a shift in carbon utilization and metabolic strategy during prolonged interaction, as previously observed in *Neurospora crassa* (Xie et al., 2004). Collectively, these studies demonstrate that MHB orchestrate a progressive and stage-dependent reprogramming of fungal gene expression, coordinating cellular remodeling, polarized growth, chromatin organization and metabolic adaptation to sustain mycelial expansion.

#### 3.3.2 Growth promotion mediated by bacterial metabolites

Beyond transcriptional reprogramming, MHB promote mycelial expansion through the production of diffusible metabolites that modulate fungal growth and development, independently of physical contact. Evidence for the activity of diffusible bacterial metabolites was provided by Schrey et al. (2007), who demonstrated that culture filtrates of *Streptomyces* sp. AcH505 were sufficient to promote hyphal growth of *A. muscaria* in a contact-independent dual culture system. Exposure to bacterial metabolites induced pronounced morphological changes, including reduced hyphal diameter and enhanced apical extension, characterized by a transition from a compact actin cap to a more dispersed structure. Interestingly, northern blot analyses of the actin-encoding genes AmAct1 and AmAct2 showed no significant differences in transcript levels between axenic hyphae and those treated with bacterial filtrate. These findings indicate that unidentified diffusible bacterial metabolites modulate fungal polarity and cytoskeletal organization through post-transcriptional or post-translational mechanisms. These observations identify cytoskeletal plasticity as an early target of MHB-mediated mycelial expansion. Subsequent studies have identified several bacterial metabolites that directly contribute to fungal growth and development. One of the best-characterized examples is auxofuran, a fungal growth-promoting secondary metabolite produced by *Streptomyces* sp. AcH505. Auxofuran stimulates hyphal growth of *A. muscaria*, and enhanced mycelial extension correlates with increased ectomycorrhiza formation on spruce roots. Its production is significantly increased during bacterial–fungal cocultivation, indicating that metabolite production is influenced by the interaction between the two partners (Riedlinger et al., 2006).

Another diffusible metabolite involved in fungal growth promotion is thiamine (vitamin B1), released by *P. fluorescens* BBc6R8 (Deveau et al., 2010), as well as by *Bacillus subtilis* (Jiang et al., 2018; Abeysinghe et al., 2020). Thiamine supports fungal vegetative development by acting as a cofactor in primary metabolic pathways (Napitupulu, 2025). In addition, bacterial production of indole-3-acetic acid (IAA) has been associated with stimulation of fungal growth. Auxin-producing bacteria, including species of *Arthrobacter* and *Pseudomonas*, enhance hyphal branching and facilitate fungal ingress into host roots, thereby promoting ectomycorrhizal establishment (Navarro-Ródenas et al., 2016). VOCs represent an additional class of bacterial metabolites capable of influencing fungal evolution over relatively long distances, although most available evidence derives from non-mycorrhizal fungal systems. For example, VOCs produced by *Paenibacillus peoriae* M48F stimulated hyphal extension in several *Pleurotus* species, with 2,5-diisopropylpyrazine identified as one of the principal bioactive compounds (Orban et al., 2023). Persistent bacterial exposure also induced the production of the fungal volatile β-bisabolene, possibly as a defense reaction. Similarly, 2,5-diisopropylpyrazine promoted hyphal branching in *L. rufus* (Aspray et al., 2013).

Although these observations support a role for VOCs in fungal development, whether similar mechanisms operate during MHB–mycorrhizal fungus interactions remains unknown. While several growth-promoting compounds have been identified, the chemical identity and mode of action of many MHB-derived metabolites remain unresolved, highlighting an important direction for future research.

### 3.4 Modulation of root colonization and nutrient acquisition by MHB

Root colonization represents the transition from free-living fungal growth to intimate association with the host plant, leading to the establishment of functional mycorrhizal symbiosis. Successful colonization requires coordinated regulation of fungal development, host receptiveness, and bacterial activities. At this stage, MHB contribute to successful root colonization by modulating fungal enzymatic activities, transcriptional programs and reciprocal nutrient exchange strategies that collectively enhance symbiotic competence (**Table 4**). By promoting both efficient root colonization and the establishment of functional nutrient exchange, MHB ultimately contribute to the performance and persistence of the mycorrhizal association.

**Table 4:** Impact of MHB on root colonization and nutrient acquisition.

Mycorrhizal fungi	Mycorrhizal type	Bacteria involved	Modalities of interaction	Reported effects	References
*Endogones* spp. (*Glomeromycota*)	AMF	*Pseudomonas* spp.	Diffusible metabolites	Enhanced fungal root penetration and establishment of mycorrhizal interactions	(Mosse, 1962)
*Amanita muscaria*	EcMF	*Streptomyces* spp. AcH505	Transcriptional reprogramming	Upregulation of fungal nitrogen, sulphur and carbon metabolism pathways associated with nutrient acquisition efficiency	(Schrey et al., 2005)
*Tuber magnatum*	EcMF	*Bradyrhizobium* spp.	Nitrogen fixation	Evidence of active bacterial nitrogen fixation associated with fungal development	(Barbieri et al., 2010)
*Hymenochaete* sp. Rl	EcMF	*Bacillus pumilus* HR10	Transcriptional reprogramming	Modulation of gene expression involved in cell wall remodeling, substrate degradation and nutrient uptake	(Wang et al., 2022)
*Oidiodendron maius*	ErMF*	*Paenarthrobacter nicotinovorans*, *Bacillus circulans*	Enzymatic stimulation	Increased chitinase, glucanase and polygalacturonase activities, coupled with enhanced nutrient mobilization and stress tolerance traits	(Yang et al., 2023)

*Ericoid Mycorrhizal Fungus.

Experimental evidence describing the effects of MHB on root colonization and nutrient acquisition. The table summarizes the mycorrhizal fungal species, bacterial partners, interaction modalities, reported biological effects, and the corresponding references.

#### 3.4.1 Enzymatic remodeling and transcriptional regulation during root colonization

During mycorrhizal establishment, fungi secrete several cell wall-degrading enzymes, which facilitate hyphal penetration and subsequent root colonization (Mosse, 1962; Yang et al., 2023). In parallel, MHB can promote root colonization by modulating the production of these fungal enzymes and altering the host defense response through diffusible metabolites (Lehr et al., 2007). Early evidence provided by Mosse (1962) showed that *Pseudomonas* spp. facilitated fungal penetration of root tissues through diffusible compounds, modifying plant cell wall plasticity and thereby promoting the establishment of mycorrhizal symbiosis. Similarly, Yang et al. (2023) identified *Paenarthrobacter nicotinovorans* and *Bacillus circulans* from the rhizosphere soil of *Vaccinium uliginosum* as putative MHB that significantly increased chitinase, β-1,4-glucanase and polygalacturonase activities in *Oidiodendron maius*, an ericoid mycorrhiza in *Vaccinium uliginosum*. Wang et al. (2022) showed that *Bacillus pumilus* HR10 promoted the growth of *Hymenochaete* sp. Rl and modulated the expression of fungal genes related to cell wall remodeling (β-1,3-glucanase), substrate degradation (laccase), nutrient uptake (MFS transporters) and the transcriptional regulator SF3B4. These findings suggest that MHB influence not only fungal enzymatic activity but also the transcriptional profile of genes involved in colonization competence.

#### 3.4.2 Nutrient acquisition and nitrogen metabolism

Beyond the ability of mycorrhizal fungi to colonize root tissues, the successful establishment rate of symbiosis depends on efficient nutrient exchange between the fungal and plant partners. The ability to mobilize nutrients from soil minerals and organic matter, as well as to fix atmospheric nitrogen, is a well-known characteristic of several rhizosphere MHB (Frey‐Klett et al., 2007). Since nitrogen availability is a major growth-limiting factor in the rhizosphere, special attention has been extended to potential nitrogen-fixing bacteria associated with ectomycorrhizas (Deveau et al., 2018). Nevertheless, the functional role of the entire mycorrhizal complex in mineral and nutrient uptake processes has only recently begun to be explored and to date very little is known about the molecular mechanisms and signaling pathways that can promote the beneficial associations under specific nutrient-deficient conditions (López-Ráez et al., 2026). Schrey et al. (2005) provided early molecular evidence that *Streptomyces* sp. AcH505 stimulated expression of fungal genes involved in nitrogen metabolism (e.g., glutamine synthetase), sulphur metabolism (adenylyl sulphate kinase, sulphite reductase), and carbon metabolism in the ectomycorrhizal fungus *A. muscaria*. These findings suggest that MHB enhance fungal nutrient acquisition by transcriptionally reprogramming fungal metabolic pathways rather than by directly supplying nutrients. Consistently, Barbieri et al. (2010) demonstrated active nitrogen fixation within *Tuber magnatum* ascomata, providing the first molecular and biochemical evidence in truffles that diazotrophic bacteria associated with truffles contribute to nitrogen availability during fungal life-cycle. These findings support the hypothesis that diazotrophic bacteria contribute to nitrogen availability during fungal development and may represent an important nutritional component of the mycorrhizal symbiosis. Collectively, these findings indicate that MHB not only facilitate fungal access to host roots but also enhance the functional competence required to establish and maintain a functional mycorrhizal symbiosis.

## 4 Ecological and applied perspectives

Despite the growing body of research on MHB, our current understanding of their ecological roles, mechanisms of action, and evolutionary significance remains fragmentary. The concept of MHB has evolved from a purely descriptive term referring to bacteria that enhance mycorrhization, to a more comprehensive framework encompassing a wide range of beneficial interactions between fungi and bacteria. However, the definition itself still lacks precision, often overlapping with that of PGPB; this necessitates a clearer functional classification based on molecular and ecological criteria. Recognizing the profound impact of these bacterial communities on the interdependent bacterium–fungus–plant holobiont, their potential application to mitigate the current climate crisis and sustain agricultural productivity under rapid population growth is currently under intense scrutiny.

### 4.1 MHB in forestry

Particularly intriguing is the role that MHB could play as drought-alleviating symbionts, enhancing the ability of mycorrhizal fungi and associated plants to thrive in arid environments. Reis et al. (2021) observed that a consortium composed of *Russula* spp. and Burkholderaceae consortium could promote cork oak (*Quercus suber* L.) survival in arid Mediterranean climates, actively fostering oak forest resilience. The same study revealed how certain *Streptomyces* species can promote the growth and proliferation of *A. muscaria* and *Suillus bovinus* mycelium, while simultaneously inhibiting *Hebeloma cylindrosporum*. This inhibition occurs through the release of rhizosphere compounds of an antibiotic nature, to which *A. muscaria* and *S. bovinus* exhibit inherent resistance (Reis et al., 2021). This observation suggests that MHB may reflect contrasting ecological strategies toward different fungal partners, selectively reshaping ectomycorrhizal community structures and suppressing competing or non-compatible organisms within the same niche (Frey‐Klett et al., 2007). Deciphering these selective antagonistic behaviors could foster the development of eco-friendly alternatives to soil fumigation and synthetic fungicides, thereby supporting plant growth under varying climate conditions. However, due to the partial overlap between MHB and PGPB (both taxonomically and functionally), precisely quantifying the direct contribution of the bacterial symbiont to plant fitness, especially as compared to the beneficial action performed by the fungal counterpart, remains difficult. This further confirms the intricate nature of the relationship established within the bacterial-fungal-plant continuum, demonstrating the strong interdependence between the different groups. Another promising area where the characterization of MHB holds significant economic value is related to truffle harvesting and commerce. Truffles represent the hypogeous fruiting body of fungi belonging to the genus *Tuber* (*Ascomycota* phylum) (Amicucci and Ranocchi, 2024), highly prized ectomycorrhizal fungi, which, alongside their “core microbiota”, host a wide range of potential symbiotic partners. Considering that this accessory bacterial microbiome is strongly influenced by the specific harvesting site and environmental conditions, MHB have been proposed as feasible “tracer microorganisms” to guarantee the geographic traceability of commercialized truffles (Monaco et al., 2022).

### 4.2 MHB contribution to nutrient cycling and stress adaptation

When stable connections have been established, both partners are involved in a mutualist symbiotic relationship, characterized by a reciprocal nutrient exchange. The bacterial counterpart receives carbon compounds from the fungus in exchange for sustaining fungal growth, nutrient uptake, and yield (Frey‐Klett et al., 2007). In this biological system, MHB exhibit a plethora of biochemical pathways capable of improving their hosts’ fitness, ranging from mineral and nutrient mobilization to antibiotic production and reduced soil stress. Among nutrients and minerals, nitrogen, phosphorus and iron are valuable macronutrients and essential to sustain fungal and plant growth. Although nitrogen is essential to sustain fungal growth, atmospheric dinitrogen (N_2_) cannot be directly assimilated by fungi or plants. This limitation is overcome by several bacterial genera, such as *Rhizobium*, *Bradyrhizobium*, *Sinorhizobium*, *Allorhizobium*, *Azorhizobium*, *Mesorhizobium*, *Frankia*, *Azoarcus*, *Achromobacter*, *Burkholderia* and *Herbaspirillum* (Olanrewaju et al., 2017). These taxa utilize a highly reducing enzymatic complex, the nitrogenases, encoded by 20 genes organized in seven *nif* operons. Fungus-associated diazotrophs, therefore, are responsible for a sensible input of organic nitrogen into forest ecosystems, especially in temperate and boreal forests (characterized by cold and acidic soil), where nitrogen is usually segregated in recalcitrant humic compounds or associated with carbon to form heavy, poorly available compounds (Frey‐Klett et al., 2007). Alongside nitrogen, phosphorus represents another abundant yet inaccessible mineral in the soil ecosystem, as it predominantly occurs in insoluble forms (organic and inorganic) not readily accessible to plants and fungi (Glick, 2012). However, the fungal ability to solubilize phosphorus seems to be enhanced by its bacterial symbionts, especially due to the formation of mixed bacterial-fungal biofilm scavenging structures, granting greater nutrient mobilizing capacity to both partners (Frey‐Klett et al., 2007).

Iron, in its ferric form (Fe^3+^), is the fourth most abundant element on earth, yet its assimilation is perilous, both for plants and microorganisms, leading to significant inter and intra-specific competition to allocate sufficient concentrations to sustain growth and differentiation (Glick, 2012). Several MHB produce siderophores, a class of molecules specialized in iron chelation, facilitating its uptake by associated fungi (Olanrewaju et al., 2017). Considering the scarce accessibility to iron in soil and the assimilation activity mediated by bacterial siderophores, fungal partners benefit from mutualistic partnerships with siderophore-producing bacteria. Additionally, such high levels of affinity to iron can reduce both biotic and abiotic stress in symbiotic partners: primarily by acting as a means of population control against pathogenic microbes, by reducing Fe^3+^ availability and stocking it within fungal and plant symbionts, causing a decline in pathogen proliferation (Olanrewaju et al., 2017). These compounds could potentially mitigate biotic stress in their partner symbionts. Collectively, these findings highlight the function of MHB not only as modulators of fungal physiology, but also as major contributors to nutrient cycling, microbial community dynamics and ecosystem resilience, reinforcing their ecological role.

### 4.3 MHB in agriculture

Current intensive agricultural practices heavily rely on the use of chemical fertilizers to augment crop yield, influencing the physicochemical and biological properties of natural soils. The main outcome is an accelerated acidification of soils, intended to facilitate nutrient uptake by plants; however, the prolonged use of such compounds can lower the pH of the soil to the extent of increasing the solubility of metals (such as aluminum and manganese), reaching toxic levels for plants (Dikir, 2023). Concurrently, synthetic fertilizers showcase scarce efficiency, due to physiological limits in plant nutrient uptake and the high solubility displayed by these chemical compounds, causing copious amounts of fertilizer to be leached by the soil (Dikir, 2023). It appears evident that these practices represent a significant waste of resources, since plants are incapable of fully benefiting from such dispendious procedures. Moreover, the long-term effects associated with the protracted usage of chemical fertilizers have already been (partly) documented: contaminated groundwater sources, eutrophication, hardpan formation (hard layer of soil, born of clay and fertilizers interaction), loss of bacterial biodiversity and intensified soil friability, drastically reducing air circulation (Dikir, 2023). Considering that recent projections by FAO (Food and Agriculture Organization of the United Nations) estimate the global human population will approach 9 billion individuals by 2050 (Nasslahsen et al., 2022), the development of novel, regenerative agricultural strategies has become imperative. Thus, deciphering the mechanisms of interaction guiding this three-way symbiosis could potentially elucidate MHB’s role as biofertilizers. In this regard, trials conducted both in the greenhouse and field demonstrated the advantageous effect of MHB and fungal co-inoculation on crops, namely by reducing the need for fertilizer administration by 10-30% and 5-10%, respectively (Guan et al., 2026). Although these estimates are not sufficient to withstand a complete revolution of the agricultural system, the rapid progress and increased research focus witnessed during the last few years appear promising to overcome the impediments encountered thus far. The main constraints to widespread MHB usage as biofertilizers lie in the nature of the bacterial carrier and their current legislation: to deliver the bacterial cells successfully, various formulations have been developed ranging from solid to liquid and even encapsulating carriers (Cárdenas-Flores et al., 2025). The limitations vary according to the modality of delivery of the bacterial symbionts, mainly regarding uniformity of distribution in soil, inconsistent field performance (aggravated by heavy-metal, drug and agrochemical pollutants), shelf life and high production costs, as well as uncertainty on the by-product effects on native soil biodiversity (Cárdenas-Flores et al., 2025).

However, appropriate legislation represents a further obstacle to widespread biofertilizer use, seeing as few nations worldwide have drafted dedicated regulations and coordination efforts across the globe are still lacking (Cárdenas-Flores et al., 2025). Therefore, future studies on the long-term effects of bacterial inoculum on soil native biodiversity and other non-target organisms are necessary to ensure the adequacy of these novel technologies, concurrently with effective standardization and legislation.

### 4.4 MHB in the future

Despite the substantial progress made in characterizing MHB (Garbaye, 1994; Frey‐Klett et al., 2007; Deveau and Labbé, 2016), the evidence synthesized throughout this review raises the possibility that fungal development is regulated by temporally distinct bacterial partners acting at successive stages of the fungal life cycle. The stage-dependent responses described across spore germination, presymbiotic hyphal development, mycelial expansion, root colonization, and nutrient acquisition suggest that MHB should not be regarded as a functionally homogeneous group of growth-promoting bacteria. Instead, they may represent a dynamic network of bacterial partners whose regulatory functions change according to the developmental requirements of the fungal host. Testing this stage-succession hypothesis should therefore become a major priority for future research. Addressing this question will require moving beyond the simplified experimental systems that have provided most of our current knowledge. Although dual culture assays and greenhouse experiments have been fundamental for identifying bacterial effects on fungal morphology and physiology (Frey‐Klett et al., 2007; Deveau and Labbé, 2016), they cannot fully capture the temporal and ecological complexity of natural mycorrhizal systems. Future studies should therefore adopt a stage-resolved approach, following the fungal life cycle while simultaneously characterizing the composition and functional activity of associated bacterial communities. Such an approach would help determine whether the same bacterial taxa perform multiple functions throughout fungal life-cycle or whether distinct MHB communities are sequentially recruited to regulate specific developmental transitions.

Resolving these questions will depend on the integration of complementary experimental approaches. Multi-omics strategies, including metagenomics, metatranscriptomics, proteomics, metabolomics and spatial analyses of microbial interactions, will be essential to identify the regulatory networks, signaling molecules and metabolic exchanges underlying bacteria as modulators of fungal progression. Attention should be devoted to deciphering the molecular mechanisms coordinating transcriptional reprogramming, metabolite-mediated communication, cell wall remodeling, and nutrient exchange across successive developmental stages. At the same time, expanding investigations beyond arbuscular and ectomycorrhizal model systems will be important to determine the extent to which these regulatory mechanisms are conserved across different mycorrhizal types and environmental contexts.

Finally, translating mechanistic knowledge into practical applications will require validating these interactions under natural environmental conditions, where fungal life is shaped by highly complex microbial communities and fluctuating abiotic factors (Nasslahsen et al., 2022; Guan et al., 2026). Rather than focusing on single bacterial inoculants, future strategies may benefit from designing functionally complementary microbial consortia capable of supporting different stages of fungal development. Such a mechanistic and stage-oriented perspective may ultimately provide the conceptual basis for more predictable microbiome engineering approaches aimed at improving mycorrhizal establishment, plant performance, and ecosystem resilience in sustainable agriculture and forestry.

## 5 Conclusions

MHB have traditionally been described as microorganisms that enhance mycorrhization or stimulate fungal growth. By integrating evidence across successive stages of fungal life-cycle, this review proposes a broader conceptual framework in which MHB are viewed as dynamic regulators of fungal development programs rather than as generic growth-promoting bacteria. From the transition of spores from dormancy to active growth, through presymbiotic hyphal development and mycelial expansion, to root colonization and nutrient acquisition, the available evidence indicates that bacterial partners influence fungal physiology through coordinated mechanisms involving transcriptional reprogramming, metabolite-mediated signaling, enzymatic activities, and other emerging regulatory processes. Although the molecular basis of many of these interactions remains only partially understood, the stage-dependent perspective adopted here helps reconcile the apparent functional diversity of MHB reported across different fungal species, bacterial taxa, and environmental conditions. Rather than representing contradictory observations, these variable outcomes likely reflect the dynamic nature of bacterial regulation throughout the fungal life cycle. Viewing MHB through this developmental framework not only provides a more coherent interpretation of existing knowledge but also establishes new directions for future research, emphasizing the need to investigate bacterial functions as temporally regulated components of fungal development. In conclusion, reframing MHB as coordinators of the fungal life cycle provides a solid conceptual foundation for guiding future research and for designing targeted microbiome engineering strategies aimed at improving mycorrhizal establishment, sustainable agriculture, forestry, and ecosystem resilience under changing environmental conditions.
